# Prevention of contamination by biopsy needle track contamination using a novel adriamycin-loaded gelatin sponge

**DOI:** 10.1186/1477-7819-11-169

**Published:** 2013-07-27

**Authors:** Zhen-Feng Li, Jian-Min Li, Jun Yan, Zhi-Ping Yang, Xin Li, Qiang Yang

**Affiliations:** 1 From the Department of Orthopaedics, Qilu Hospital of Shandong University, 107 Wenhuaxilu, Jinan, Shandong Province 250012, China

**Keywords:** Adriamycin, Gelatin sponge, Biopsy track, Embolism, Osteosarcoma

## Abstract

**Background:**

The gold standard of tumor diagnosis is histological examination of a biopsy; however, there is concern that tumor cell dissemination along the needle track during percutaneous biopsy can cause local tumor relapse. We aimed to evaluate the value of an adriamycin (ADM)-loaded gelatin sponge in preventing tumor cell contamination along the biopsy needle track.

**Methods:**

Data were obtained from 40 patients who were diagnosed by core needle biopsy as having osteosarcoma and who were followed up at our hospital between 2008 and 2011. Of the 40 patients, 20 had the needle biopsy tracks filled with ADM-loaded absorbable gelatin sponge immediately after the biopsy specimen was obtained, while the other 20 did not. All 40 patients underwent limb-salvage surgery, and specimens were obtained from the biopsy track for histopathologic examination of multiple sections.

**Results:**

On histological examination, there was less tumor cell contamination along the biopsy tracks in the ADM group.

**Conclusion:**

Use of ADM-loaded absorbable gelatin sponge may prevent tumor cell contamination of a biopsy track, and reduce the possibility of consequent tumor relapse.

## Background

The gold standard of tumor diagnosis is histological examination of a biopsy. Core needle biopsy is becoming the main method of obtaining a sample for pathologic examination for diagnosis of osteosarcoma; however, there is concern that tumor cell dissemination along the needle track during percutaneous might cause local tumor relapse. A few recent reports indicated that tumor cell contamination [1] and local metastasis [2-4] did occur along the needle puncture track. To decrease the likelihood of tumor cell contamination along the track during core needle biopsy, we designed a novel adriamycin (ADM) loaded gelatin sponge and use this to embolize the biopsy needle track. Specimens from the biopsy track were later obtained, and the pathologic results were evaluated and compared with the specimens of biopsy track. The histological examination showed that there was less tumor cell contamination along the biopsy tracks in the ADM-treated group.

## Methods

During the period 2008 to 2011, 40 patients with suspected osteosarcoma underwent core needle biopsy. Before biopsy, the 40 patients were randomly divided using a digital table into the ADM (experimental) and non-ADM (control) groups, with equal numbers of patients in each. Details of the clinical data of both groups are illustrated in Table 1.

**Table 1:** Clinical data of the groups

**Characteristic**	**ADM group**	**Non-ADM group**
Age, years	21 (10 to 29)	23 (12 to 30)
Tumor site		
Femur	9	11
Tibia	7	9
Humerus	2	0
Scapula	1	0
Enneking grade		
IIA	13	12
IIB	7	8
Biopsy		
Needle	20	20
Open	0	1
Type of surgery		
Limb salvage	17	17
Amputation	3	3
Pre-operative chemotherapy		
2	17	18
1	0	2
0	3	0

**Figure 1:**
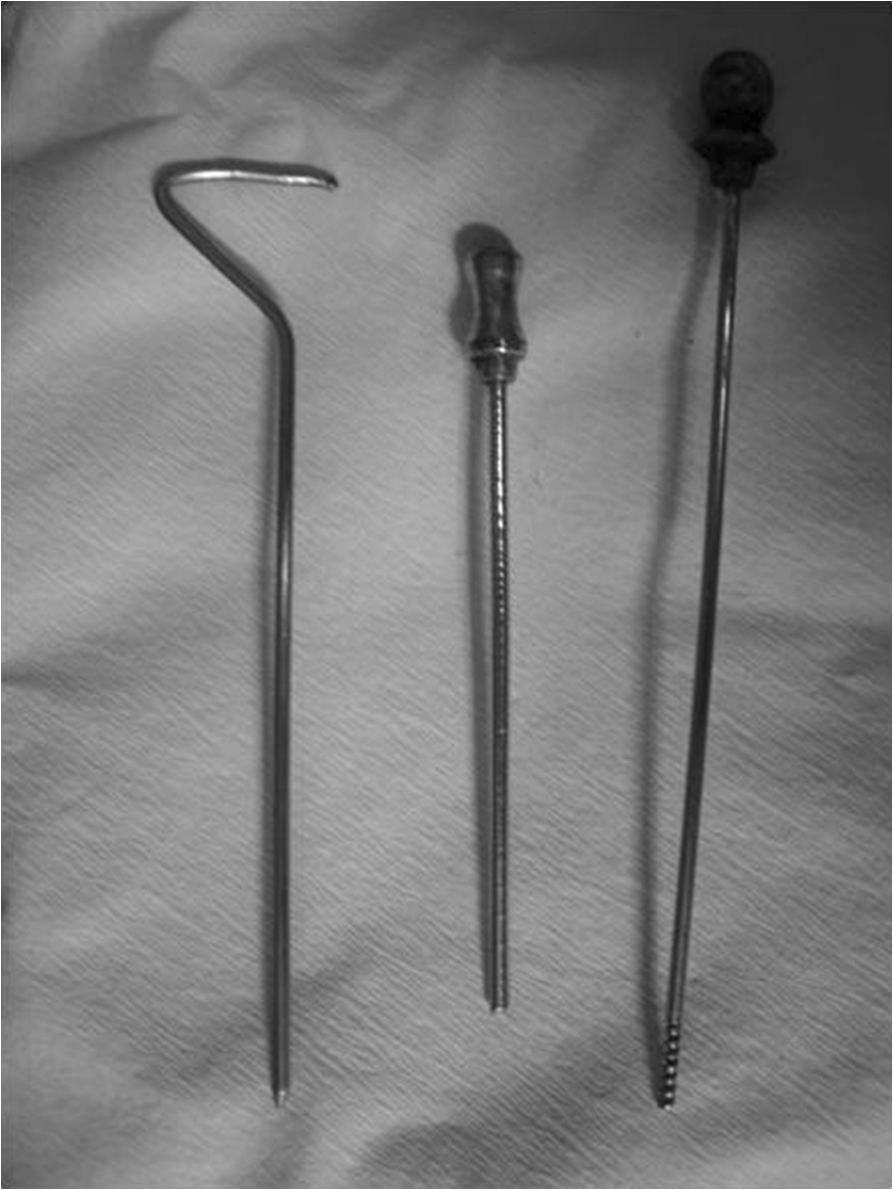
The puncture needle: the inner needle, outer cylindrical cuff and spring-loaded cutting needle.

We used a homemade spring-loaded cutting needle to perform the biopsy (Figure 1). This unique spiral-blade needle was in the shape of a spiral screw, with which tumor tissue could be obtained. The biopsy method was as follows. After local anesthesia was administered, the skin over the biopsy site was punctured with the biopsy needle. The inner needle was then withdrawn from the outer cylindrical cuff and changed to a spring-loaded cutting needle through the outer cylindrical cuff. Once the surface of the tumor was reached, the cutting needle was slowly rotated forward into the tumor mass in a clockwise direction, resulting in the screw groove of the spring-loaded cutting needle being filled with tumor tissue. The spiral blade was then withdrawn, and the biopsy tissue was removed from the needle. By repeating these steps, tumor samples at different depths and sites could be collected. The tumor samples were then sent for pathologic examination.

**Figure 2:**
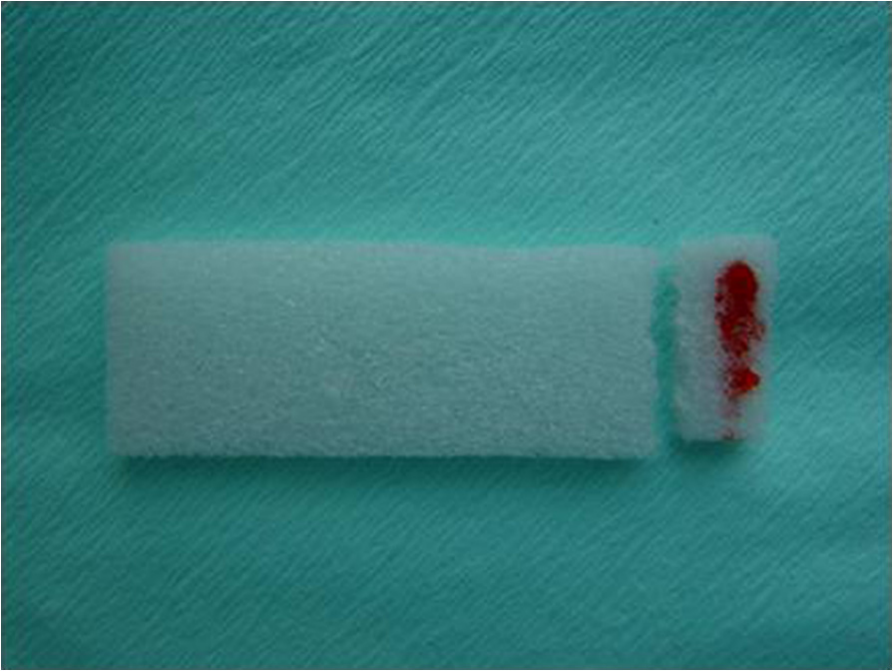
**Gelatin sponge with 1 mg adriamycin (ADM).** The ADM is visible as a red color on the sponge.

After a satisfactory tumor sample was obtained with the spiral needle, the experimental (ADM) group was treated with ADM. A gelatin sponge containing 1 mg ADM (prepared from a bottle containing 10 mg ADM, diluted with 1 ml normal saline, and 0.1 ml used) was inserted into the track through the working tunnel, until it reached the surface of the tumor mass (Figure 2). A second piece of gelatin sponge then inserted through the outer cylindrical cuff to a point about 0.5 cm above the tumor mass capsule; this sponge did not contain ADM, in order to prevent the spread of the drug to healthy tissue.

For the control group, no gelatin sponge was used to fill the biopsy track, the biopsy syringe was withdrawn immediately after the biopsy was taken, and the biopsy site was compressed with a dressing.

The histopathology confirmed that all 40 biopsy specimens were osteosarcoma, and all 40 patients had limb-salvage operations. During the surgery, the needle tracks were resected *en blo*c with the tumor mass, and the biopsy-track specimens were separated from the tumor mass for pathologic examination. The measured distances between the skin puncture and tumor was about 2.5 cm (range 1.5 to 3.2 cm), and the needle track diameter was about 3 mm. The specimens were fixed in formalin and cut sections into tissue blocks with the 2 mm spacing paralleling the needle within 1 cm distance to the puncture track, and the tissue blocks were cut sections 6 μm thick. All specimens from both groups were evaluated histologically by an experienced pathologist.

## Results

All biopsy procedures were successfully performed. No acute complications, such as skin necrosis, infection, or damage to blood vessels or nerves were noted after biopsy. The punctured needle holes healed uneventfully in all cases.

The tumor cell necrosis rates of tumor mass specimens in the ADM and non-ADM groups were 8/20 and 9/20, respectively for higher grades (Huvos III/IV >90%), and 12/20 and 11/20 for lower grades (Huvos I/II < 90%). Of the 40 patients, 37 (2 of ADM group and 1 of non-ADM group could not be contacted) had follow-up data available, and the average follow-up time was 29 months (range 5 to 42). Endpoints included the recurrence, metastasis, death and the most recent follow-up. The rates of recurrence outside the biopsy site were 5.6% (1/18) for the ADM group and 11% (2/19) for the non-ADM group; the 3-years event-free survival rates (EFS: recurrence, metastasis and death) were 61% (11/18) and 63.1% (12/19), respectively, and the overall survival (OS) rates were 66.7% (12/18) and 73.7% (14/19, respectively). There was no significant difference (*P* < 0.05) between the ADM and non-ADM groups. Outcome details are illustrated in Table 2.

**Table 2:** Patient outcome after treatment

**Characteristic**	**ADM group**	**Non-ADM group**
Tumor cell necrosis		
Huvos III, IV	40% (8/20)	45% (9/20)
Huvos I, II	60% (12/20)	55% (11/20)
3-year EFS	61% (11/18)	63.1% (12/19)
3-year OS	66.7% (12/18)	73.7% (14/19)
Non-biopsy site recurrence	5.6% (1/18)	10.5% (2/19)
Tumor cells	0/20	3/20

EFS, event-free survival; OS, overall survival.

**Figure 3:**
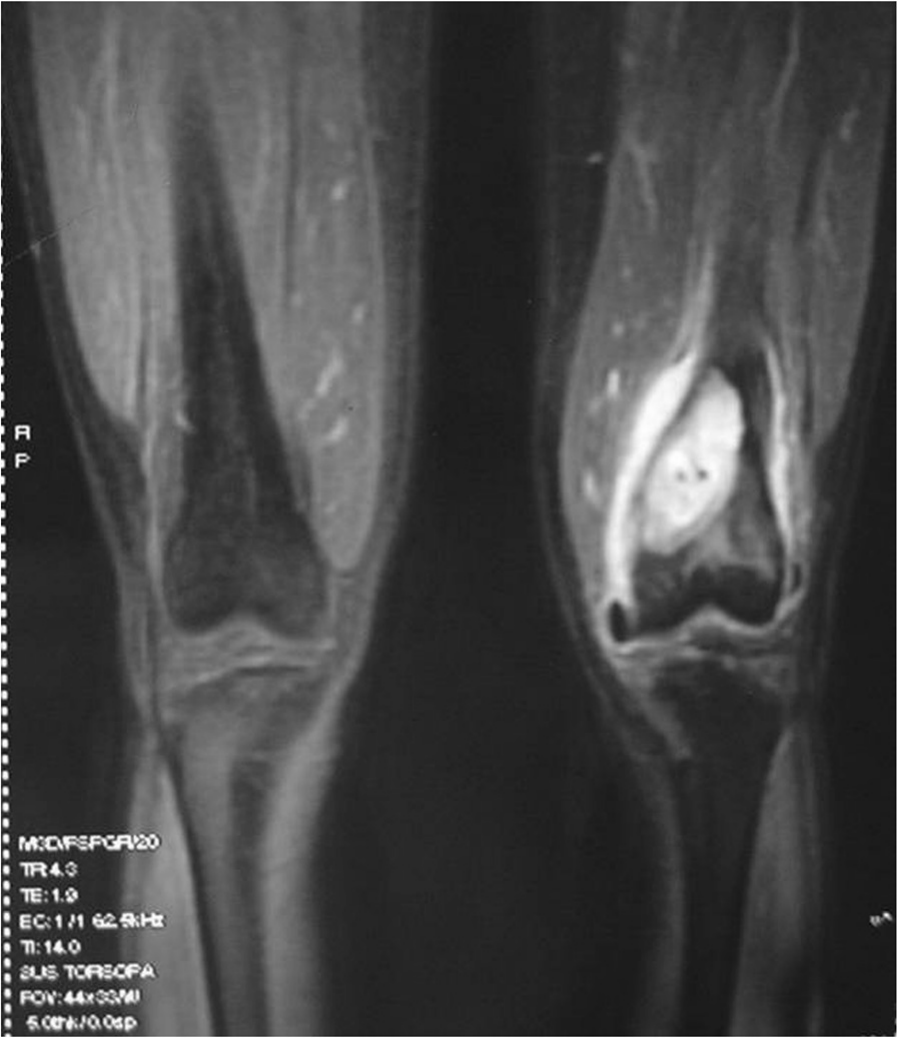
Magnetic resonance imaging scan of osteosarcoma in the control.

**Figure 4:**
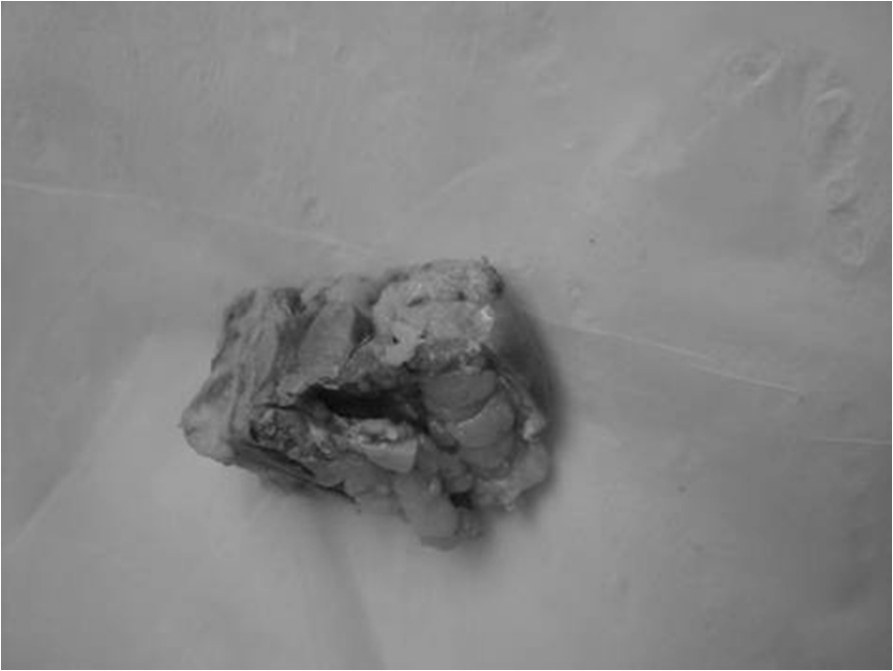
The needle track specimen.

**Figure 5:**
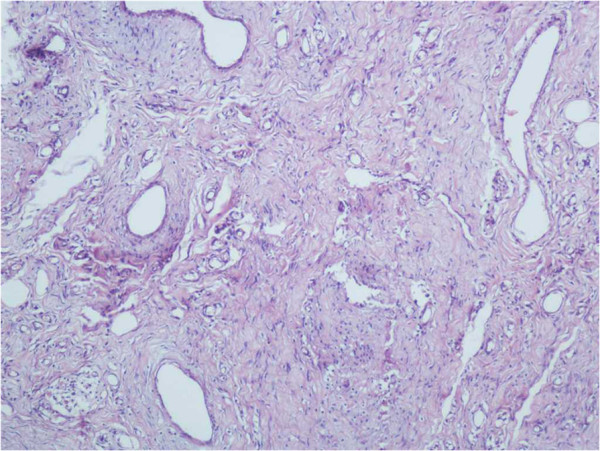
Fat and tumor cells in the pathology examination.

No tumor seeding was found along the needle tracks based on the pathologic results for the ADM group. In the control group, tumor cells were found along the needle tracks in three cases (Figures 3, 4 and 5). Using the small sample *t*-test a significant difference (*P* < 0.05) was found between the ADM and non-ADM groups.

## Discussion

Pathologic examination is the most important procedure for diagnosis of bone tumors compared with radiography, computed tomography, and magnetic resonance imaging. Biopsy methods include pre-operative open biopsy, core needle biopsy and the intra-operative biopsy. However, both the open and the intra-operative biopsy carry the risks of non-healing of incision and tumor implantation of needle track. Enneking [5] reported that the local contamination rate by tumor cells of intra-operative incision was 39%. With the development of diagnostic technology, the core needle biopsy is being used extensively because of its simple manipulation, minimal invasiveness into the adjacent tissue and reliable results [6].

In recent years, there have been reports of local dissemination of tumor cells along needle tracks. After investigating cases of local metastasis after limb-salvage surgery, one group reported that the incidence of tumor cell contamination along the biopsy needle track was about 6.6% [1] Other reports showed that local tumor cell contamination occurred after needle biopsy without biopsy-track treatment [2-4]. Schwartz [7] reported that local tumor metastasis occurred in 89% of tracks after needle biopsy of sarcoma, and there were 57 cases of metastasis along the biopsy needle track from 1950 to 1997. Consequently, it is important to find ways to reduce the incidence of local tumor cell contamination along biopsy needle tracks. Wiksell [8] reported that no tumor cells were found in fine needle tracks after treatment with radiofrequency pulses to the tracks. However, the diameters of most core needles used for bone tumors are large, exceeding 16G [6,9]. To prevent local tumor cell contamination along such needle tracks, we used ADM-loaded gelatin sponge to embolize the needle tracks after biopsy, and evaluated the outcome with pathologic examination.

During the biopsy, we obtained the tumor specimen using a spring-loaded cutting needle, within the outer cylindrical cuff. In this way, there was minimal invasiveness to the surrounding tissue, and sufficient tumor specimens could be obtained from different depths and in different directions through the same working tunnel. Furthermore, the location of the puncture point guided the position of the future surgical incision so that the needle track could be resected *en blo*c with the tumor mass during the final operation [10].

The appropriate length of follow-up is an important area of controversy [11]. The reporte recurrence rates after osteosarcoma treatment range from 2.6 to 7.9% [12,13], and the mean time to local recurrence has been variously reported as 13, 14, 18 and 27 months [12,14-17]. In this study, our mean follow-up for recurrence was 29 months. Because the study was mainly concerned with recurrence along the biopsy track, and the tracks were resected in the surgery, thus we consider that 29 months was sufficient to detect any recurrence. However, we will continue to follow up these patients.

Another controversial point is identifying whether a tumor is a recurrence or the result of tumor cell seeding [11]. In our study, we resected all biopsy tracks during the surgery. However, one recurrence site occurred near the incision, thus we could not be sure whether it was in fact a recurrence or was the result of tumor cell seeding.

We found tumor cells in the biopsy-track specimens in three patients in the non-ADM group, thus the rate of tumor cell contamination was 15% (3/20) which is similar to results from a previous report [1]. There was no significant difference in EFS and OS between the two groups. Saghieh [18] reported that limb-salvage operations could be performed safely using standard approaches without the need for biopsy-track excision, as the incidence of tumor cell seeding was believed to be low; however, that study included only 10 cases. Most other reports have recommended resecting the biopsy track to decrease the incidence of possible tumor relapse from the needle track.

ADM is a chemotherapy drug used routinely in the treatment of osteosarcoma. Its pharmacological mechanism is probably related to combining the cell DNA, then inhibiting nucleic acid synthesis and mitosis. ADM is usually administered by intravenous injection. Its cytotoxicity is proven and it is a strong vesicant leakage of ADM outside the superficial veins can lead to local tissue inflammation, swelling, skin ulcers, necrosis, and even dysfunction of limb; however, there has been no report of adverse events occurring with mild ADM leakage outside the deep veins. The cases in our study all had deep bone tumors. Above the surface of the tumor mass, there is skin, subcutaneous fat, deep fascia and muscle, which all have the effect of minimizing the local tissue damage from ADM. In our study, the ADM-loaded gelatin sponge was placed just above the surface of the tumor mass through the needle track, and the superficial part of the track was filled with a plain gelatin sponge without ADM to prevent the spread of the drug. The results of our study showed that the small dose of ADM deep located on the surface of tumor mass was able to provide a local chemotherapeutic effect without causing serious damage to the surrounding normal tissues.

## Conclusion

We consider that the use of ADM-loaded gelatin sponge during biopsy could provide a useful method for embolism in needle tracks. The ADM-loaded gelatin sponge prevented blood (possibly containing tumor cells) moving from the tumor along the biopsy track (tumor cell contamination is more likely to happen in malignant tumors with a rich blood supply), providing both an embolization function and a local chemotherapeutic effect. This method is simple, safe, and effective, and it may be particularly useful after biopsy of bone tumors with favorable soft-tissue coverage.

## Abbreviations

ADM: Adriamycin; EFS: Event-free survival; OS: Overall survival.

## Competing interests

All authors declare that they have no competing interests.

## Authors’ contributions

ZL carried out the surgery, collected data, and wrote the manuscript. JL designed the study and provided the patients. ZY and XL participated in the study design and coordination, and helped to draft the manuscript. QY and JY participated in the pathologic examinations and collected data. And JY revised the paper. All authors read and approved the final manuscript.

## Authors’ information

Each author certifies that they have no commercial associations (for example, consultancies, stock ownership, equity interest, patent/licensing arrangements, etc.) that might pose a conflict of interest in connection with the submitted article.

Each author certifies that Ethics Committee of Qilu hospital of Shandong University has approved the human protocol for this investigation, that all investigations were conducted in accordance with ethical principles of research, and that informed consent for participation in the study was obtained from all participants.
